# Opportunity cost determines free-operant action initiation latency and predicts apathy

**DOI:** 10.1017/S0033291721003469

**Published:** 2023-04

**Authors:** Akshay Nair, Ritwik K. Niyogi, Fei Shang, Sarah J. Tabrizi, Geraint Rees, Robb B. Rutledge

**Affiliations:** 1 Huntington's Disease Centre, UCL Queen Square Institute of Neurology, University College London, Russell Square House, 10-12 Russell Square, London, WC1B 5EH, UK; 2 Max Planck UCL Centre for Computational Psychiatry and Ageing Research, UCL Queen Square Institute of Neurology, University College London, Russell Square House, 10-12 Russell Square, London, WC1B 5EH, UK; 3 Department of Psychiatry, Yale University, New Haven, CT 06510, USA; 4 Wellcome Centre for Human Neuroimaging, UCL Queen Square Institute of Neurology, University College London, 12 Queen Square, London WC1N 3AR, UK; 5 UCL Institute of Cognitive Neuroscience, UCL Queen Square Institute of Neurology, University College London, 17-19 Queen Square, London, WC1N 3AZ, UK; 6 Department of Psychology, Yale University, New Haven, CT 06511, USA

**Keywords:** Apathy, motivation, opportunity cost, reinforcement learning

## Abstract

**Background:**

Apathy, a disabling and poorly understood neuropsychiatric symptom, is characterised by impaired self-initiated behaviour. It has been hypothesised that the *opportunity cost of time* (OCT) may be a key computational variable linking self-initiated behaviour with motivational status. OCT represents the amount of reward which is foregone per second if no action is taken. Using a novel behavioural task and computational modelling, we investigated the relationship between OCT, self-initiation and apathy. We predicted that higher OCT would engender shorter action latencies, and that individuals with greater sensitivity to OCT would have higher behavioural apathy.

**Methods:**

We modulated the OCT in a novel task called the ‘Fisherman Game’, Participants freely chose when to self-initiate actions to either collect rewards, or on occasion, to complete non-rewarding actions. We measured the relationship between action latencies, OCT and apathy for each participant across two independent non-clinical studies, one under laboratory conditions (*n* = 21) and one online (*n* = 90). ‘Average-reward’ reinforcement learning was used to model our data. We replicated our findings across both studies.

**Results:**

We show that the latency of self-initiation is driven by changes in the OCT. Furthermore, we demonstrate, for the first time, that participants with higher apathy showed greater sensitivity to changes in OCT in younger adults. Our model shows that apathetic individuals experienced greatest change in subjective OCT during our task as a consequence of being more sensitive to rewards.

**Conclusions:**

Our results suggest that OCT is an important variable for determining free-operant action initiation and understanding apathy.

## Introduction

Apathy is a common, disabling and hard-to-treat neuropsychiatric symptom found in a range of neuropsychiatric disorders such as schizophrenia, depression, Parkinson' disease (PD), Alzheimer' disease (AD) and Huntington' disease (HD) (Husain & Roiser, 2018; Krishnamoorthy & Craufurd, 2011; Le Heron, Apps, & Husain, 2018). In these populations, clinical apathy is commonly associated with reduced self-care, functional decline and the need for external support (Konstantakopoulos et al., 2011; Pagonabarraga, Kulisevsky, Strafella, & Krack, 2015; Starkstein, Jorge, Mizrahi, & Robinson, 2006; Van Duijn, Reedeker, Giltay, Roos, & Van Der Mast, 2010). In the non-clinical population, apathy is thought to affect academic performance, productivity and health-related outcomes like weight control and later-life frailty (Ang, Lockwood, Apps, Muhammed, & Husain, 2017; Ayers et al., 2017; Desouza et al., 2012; Katzell & Thompson, 1990). Despite the prevalence and significance of apathy, it remains poorly understood. Apathy is characterised by reduced motivation and impaired self-initiated goal-directed behaviour (Le Heron, Apps, & Husain, 2018; Levy & Dubois, 2006; Marin, 1991; Starkstein, 2000). In part, our limited understanding of apathy may reflect limited understanding of a key component of ecological behaviour at the heart of apathy – self-initiation. If we better understood the environmental and computational mechanisms which drive self-initiation, can we better understand apathy?

Reinforcement learning (RL) is a prominent theoretical framework that has been used extensively to build computational models of animal and human decision making and motivation (Chowdhury et al., 2013; Garrison, Erdeniz, & Done, 2013; Huys, Maia, & Frank, 2016; Niv, Daw, Joel, & Dayan, 2007; Noonan, Kolling, Walton, & Rushworth, 2012; Pessiglione, Seymour, Flandin, Dolan, & Frith, 2006; Rutledge, Dean, Caplin, & Glimcher, 2010; Schultz et al., 1997; Voon et al., 2010). Despite the extensive use of RL to model trial-by-trial behaviour, there have been limited attempts to extend this framework to the study of self-initiated, or free-operant, behaviour. Niv et al. (2007) began to address this theoretical gap by considering the choice of free-operant action initiation latency as an optimal decision-making problem. They framed the problem of action initiation as a semi-Markov decision process and used a branch of RL known as ‘average reward’ RL to model free-operant action initiation in animals (Mahadevan, 1996; Niv et al., 2007; Puterman, 2005; Sutton, Precup, & Singh, 1999). In their influential computational model, the decision maker chooses not only which action to pick but when to take their next action. Niv et al. (2007) argued that the decision maker must have computed the average ‘reward rate’. This variable encodes the amount of reward, on average, that can be extracted from the environment, per unit time. This allows the decision maker to calculate the amount of reward which could be lost if action initiation is delayed – put simply, the cost of sloth. By weighing this ‘opportunity cost of time’ (OCT) against the energetic or ‘vigour cost’ of acting too rapidly, the decision maker can derive an optimal latency that maximises their net rewards over a period. Prompts are not required to engender action as, immediately after the last action is completed, the decision maker begins to accrue opportunity cost, which drives them to act again. In this model, the OCT is also governed by an animal' motivational status. For example, hungry animals have been shown to complete non-rewarding actions faster, such as grooming (Dickinson & Balleine, 2002; Hull, 1943; Niv, Daw, & Dayan, 2005). Within the OCT framework this is predicted as hunger increases the utility of food: penalising time spent away from seeking food. Thus, the OCT theory outlines a theoretical framework for understanding both self-initiation and motivation. Despite such insight, although this model has been applied to trial-based cognitive tasks (Beierholm et al., 2013; Guitart-Masip, Beierholm, Dolan, Duzel, & Dayan, 2011), there is currently limited evidence to suggest that in a free operant setting, healthy participants choose action latencies based on the OCT. Furthermore, the relationship between apathetic symptoms and sensitivity to OCT has not been explored. In this study, we seek to address these lacunae. It should be noted that although we approach this problem from a RL perspective, the OCT theory considerably overlap with the ‘neuroethological’ approach derived from the foraging literature which makes similar predictions (Pearson, Watson, & Platt, 2014; Stephens & Krebs, 1987).

We developed a novel behavioural paradigm in which participants were free to choose when to self-initiate actions while we experimentally manipulated the OCT. First, we predicted that in this free-operant setting, participants would rapidly adapt their choice of action latencies based on the OCT. Higher levels of opportunity cost would encourage more frequent action initiation. Second, as described above, we predicted that high opportunity cost would invigorate the completion of non-rewarding actions. Finally, we asked whether sensitivity to the OCT within our task predicted behavioural apathy scores. We hypothesised that motivated individuals would perceive even small rewards as highly rewarding. They would perform tasks as if there was a higher degree of opportunity cost throughout the task. As such, when exposed to a task with fluctuating levels of opportunity cost, motivated individuals would consistently act quickly, showing little variation in chosen action latencies. By comparison, we predicted that apathetic individuals would show a strong inverse relationship between OCT and chosen action latency, choosing to go faster only when the opportunity cost is high and slowing down when it is low. Based on previous work, we fit our data using a new, average-reward, RL model and predicted that differences in reward sensitivity parameters in our model could explain the relationship between apathy and the OCT.

## Methods

### Samples

Both studies were performed before the coronavirus disease-2019 (COVID-19) pandemic. We recruited healthy participants with no known psychiatric or neurological history and who were not taking any psychotropic medication. Participants were told that they could earn up to £5 depending on their performance. Participants who felt that they struggled with motivation were encouraged to sign up to the study, but participants were not pre-screened on apathy scores. Twenty-one participants were recruited into Exp. (1). This study was approved by the UCL ethics committee (3450/002). Ninety adult participants from the Prolific online portal (https://prolific.ac/) were included in Exp. (2) [see online Supplementary Methods for details of addition inclusion and exclusion criteria for Exp. (2)]. The study was run on the Gorilla testing platform with task code written in Javascript. Participants received additional payment to ensure that hourly earnings for participation were at least £5 per hour. This study was approved by the UCL ethics committee (12 365/002). Study demographics for both studies are shown in Table 1.

**Table 1.** Demographics for participants included in the in-lab, Exp. (1), and the online studies, Exp. (2)

	Experiment 1 (*N* = 21)	Experiment 2 (*N* = 90)
Age	22.5 ± 2.9 (min: 19, max: 32)	38.6 ± 10.6 (min: 21, max: 63)
Gender (F%)	76.2%	51.7%
AMI total	1.4 ± 0.4	1.6 ± 0.5
Behavioural AMI	1.6 ± 0.9	1.6 ± 0.8
HADS total	13.7 ± 7.2	12.7 ± 6.8

Mean total Apathy and Motivation Index (AMI), behavioural AMI sub-score and total Hospital Anxiety and Depression Scores (HADS) shown (values shown as mean ± s.d.).

### Questionnaire data

Participants completed two questionnaires after finishing the task – the Apathy and Motivation Index (AMI), a validated questionnaire designed to measure apathy in the general population; and the Hospital Depression and Anxiety Scale (HADS), a brief self-reported screening tool for assessing depressive and anxiety symptoms (Ang et al., 2017; Stern, 2014). AMI is scored such that higher scores correspond to higher apathy levels. Given the focus of our experiment was on behavioural apathy, our primary outcome for these experiments was the behavioural apathy score from the AMI (bAMI), as opposed to the emotional or social apathy scale.

### Task overview

**Fig. 1.**
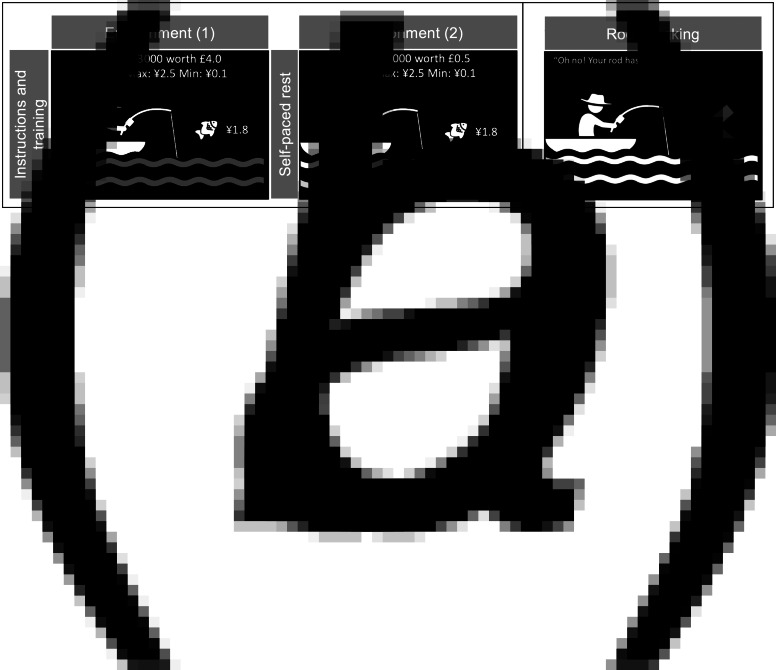
Overview of task design. (a) Following assessment of maximum tapping speed, instructions and training, participants completed two counterbalanced environments in which they earned ¥ for fish caught with key presses: high OCT and low OCT environment indicated by the monetary value of ¥3000 (£4 or £0.50) and the colour of the water (blue water representing high value and white water representing low value). When not pressing to catch fish, nothing on screen prompted action. To register that a fish was caught, the angle of the fish graphic changed by 45^o^. A bell sounded each time the price for fish changed. Information regarding environments and range of fish prices was present on screen at all times. The price of the fish changed every 12–13 s and prices were randomly drawn from a set of six prices ranging from ¥0.1–¥2.5 per fish. Each price was seen four times in an environment and the order of prices was the same in both environments but randomly generated for each participant. (b) Six times in each environment, the participant' fishing rod broke. To fix it they were required to repeatedly tap an alternative button, for no immediate reward and for a fixed number of times. While the rod was broken, no price was displayed on screen; instead, participants saw a large red cross which decreased in size with every tap. Time within the task was not stopped while the rod was being fixed and participants were aware of this.

Participants played our novel task, ‘The Fisherman Game’ shown in Fig. 1. Participants were told that they would be earning money, in fictional yen ¥, by catching fish. To catch fish, participants pressed the down arrow key on a keyboard. This action, ‘a tap’, required minimal effort and the force of tapping was not relevant to outcome. Every time they pressed down, they ‘caught’ a fish. The number of yen earned for each fish was displayed on the screen next to a fish icon. This value changed every 12–13 s and was drawn at random from a set of 6 numbers ranging from ¥0.1 to ¥2.5. When the price changed participants also heard a bell to alert them to the change in price to minimise effects of poor attention. As this task was designed to test the timing of self-initiated behaviour, the screen was static and there were no prompts to initiate actions.

Participants were told that they would play the fisherman game in two ‘environments’ each containing two blocks. One block consisted of 12 changes in price following which block participants were given a self-paced rest. Participants were told that in one environment earning ¥3000 would result in payment of £4.00. In the other environment, they were told earning ¥3000 would only result in a payment of £0.50. The order of the environments was counterbalanced between subjects, and all subjects knew before starting the game that they would have to play both environments. The change in OCT across the prices and environments represent the two OCT manipulations participants experienced in this study.

Finally, a non-rewarding action was included in both environments of the game. Participants were told that their fishing rod may break randomly during the game. To fix the rod, participants were told to tap the right arrow key on the keyboard five times successively. On the screen, rod breaking was indicated by a red cross which reduced in size with each successive tap. Rod fixing yielded no additional yen or fish and at the time the rod broke the current price of fish was not displayed, only the environment value. The only utility of fixing the rod quickly was to be able to return to collecting fish. The fishing rod broke six times per environment. When the rod broke, time in the task was not stopped and participants were aware of this task feature. The order of price changes and timings of the rod breaking were randomly determined in each participant and fixed across the two environments. The task user interface and design elements are further shown and described in Fig. 1. Before starting the experiment, participants read detailed instructions on all aspects of the task including a practice period catching fish for 20 s whilst the price changed and a period fixing the rod. They were told to go as fast or as slow as they wanted throughout the experiment. The task itself lasted approximately 15 min. The task was designed and implemented in Cogent 2000, a Matlab toolbox for psychological experiments.

The task online for Exp. 2 was almost identical to the one described above; however, the minor differences are described in the online Supplementary Methods. Additional inclusion and exclusion criteria for Exp. 2 are also found in the online Supplementary Methods. The task was coded in JavaScript and hosted on Gorilla (https://gorilla.sc/).

### Outcome measures

The key variable of interest in this study was the latency between two successive taps, calculated as the difference in the stored timestamps between two key presses. The price and the environment were manipulated as described above. Action latencies for fishing and fixing a broken fishing rod were recorded.

### Statistical analysis

Statistical analysis was identical in both studies. Basic task metrics were first computed to assess the effect of the environmental manipulation of the OCT. The number of taps were compared, within subject, between the high and low opportunity cost environments. The mean latency per price in each environment was also calculated and used to illustrate the effect of price and environment manipulation. To determine the effect of environment on rod fixing latencies, the mean log transformed rod fixing latency in each environment were compared.

Outliers were removed from latency data using median absolute dispersion technique. This outlier removal technique is itself more robust to the presence of outliers and has been recommended for use with latency data (Leys, Ley, Klein, Bernard, & Licata, 2013). Using this approach, an outlier is defined as being greater than three scaled absolute deviations from the median [median(|*Y*_i_-median(*Y*)|)]. Outliers were removed from the raw latency data for each subject in each environment. Latencies were then log transformed for use in linear regression models.

We modelled our data using a linear mixed effects model. The log transformed latencies were specified as the dependent variable in our model. At the fixed effects level, we included three variables (1) price, (2) environment (low or high as a dummy variable) and (3) the number of cumulative taps the subject had performed in the environment up to that point. Each of these effects was also estimated at the subject level as random effects. Subject level slopes for price and environment were used as individual measures of sensitivity to our two manipulations of opportunity cost. Linear mixed models were fit in MATLAB 2017a using the *fitlme* function. Models were estimated by fitting an unstructured variance-covariance matrix using a restricted maximum likelihood (REML) fit method.

We also asked whether these individual sensitivities to opportunity cost (i.e. price beta, environment beta) were predictive of bAMI. In Exp. (1), we built a linear regression model to determine whether individual sensitivity to opportunity cost predicted bAMI scores whilst controlling for demographics (age and gender) and symptoms of depression and anxiety from the HADS entered separated (D-HADS and A-HADS respectively). Exp (2), the online study had a wider age range as compared to the lab study (see Results). Based on the results from Exp. (1), post-hoc, we asked whether the effect would be present in young adults (age 18–35) and we divided our cohort in Exp. (2) into young (age 18–35) and older adults (age 36–65).

### Computational modelling

Following Niv et al. (2007) we formulated the task as a real-time cost-benefit decision-making problem, in which participants trade-off the OCT against the energetic cost of acting quickly. Formally, our approach constitutes an ‘average reward’ RL problem. We assumed that each [*price of fish, environment*] condition is a separate ‘state’. A participant when in a state, chooses a latency then returns to the same state, and the process repeats. Central to our model, participants choose action latencies (*τ*) by balancing the cost of vigour (*C*_v_/*τ*) and the OCT (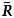 *τ*). The vigour cost is inversely proportional to the latency, with an individually fitted cost parameter (*C*_v_), accelerating rapidly as the participant responds closer toward their fastest motor latency. The OCT denotes the average reward foregone by responding at a particular latency: slower responses in a high reward environment lead to greater reward foregone. OCT is calculated by multiplying the reward rate: average reward available per second (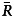), by the latency (*τ*). Aside from the *C*_v_ parameter, we also fit a reward sensitivity (*S*_R_) parameter to each subjects data. In our model, a subject with low *S*_R_ would perceive little difference in subjective rewards between prices or environments. For such a subject, the subjective reward remains high even when the price or environment value is low. By comparison, with high *S*_R_, subjective reward would relate more closely with the value of the price or environment. For more modelling details including model specification, fitting procedure and model comparison see Supplementary Methods and Fig. S1.

## Results

### Cohort description

Table 1 shows the demographic details for participants in Exp. (1) and Exp. (2). In both experiments we sought to recruit healthy adult participants. Exp. (1) took place under laboratory conditions whereas Exp. (2) was completed online using the Gorilla and Prolific testing platforms.

### Opportunity cost invigorates both rewarding and non-rewarding actions in healthy participants

**Fig. 2.**
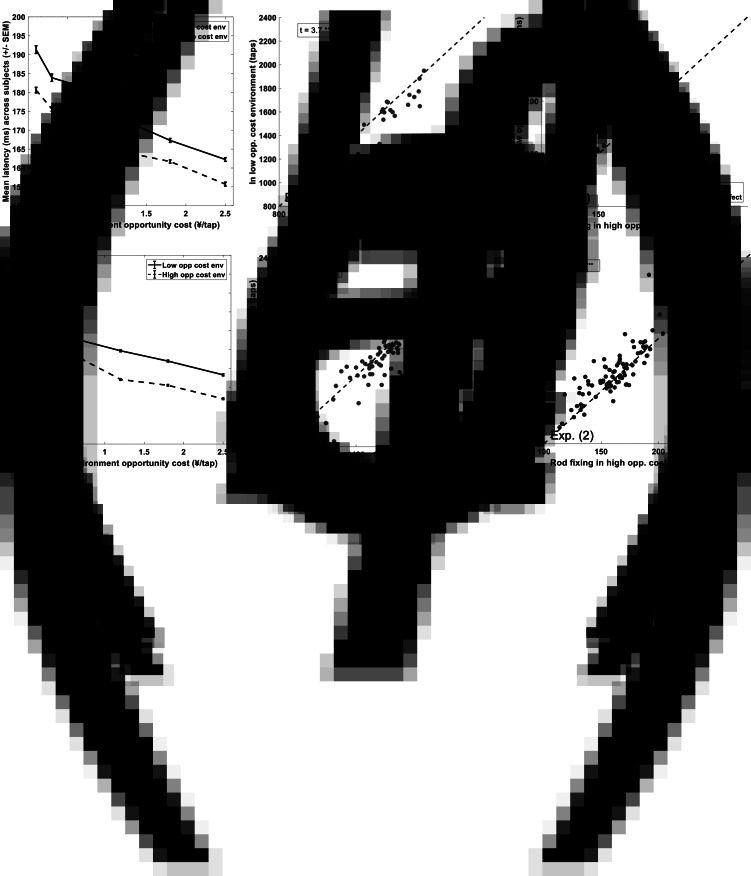
Opportunity cost invigorates rewarding actions – (a–c) show data from Exp. 1, lab-based and (d–f) show data from Exp.2, online based. (a, d) In both lab-based (a) and online (d) experiments, increased opportunity cost (manipulated by environments with a higher price for fish) produced the predicted reduction in chosen free-operant action initiation latencies. Mean choice latency is plotted by price (¥/tap) and environment (±s.e.m.) for (a) Exp. (1) in-lab sample and (b) Exp. (2) online sample. (b, e) Higher opportunity cost was associated with more frequent self-initiated action initiation (i.e. more taps) during the fixed environment duration in subjects in both (b) Exp. (1) in-lab and (E) Exp. (2) online studies. Grey dots represent the number of taps performed by each subject in each environment (low v. high opportunity cost). *t* statistic shows paired difference between number of taps. (c, f) Higher opportunity cost environments are associated with faster rod-fixing latencies, despite rod fixing being an action with no immediate reward value in both environments, in both (c) Exp. (1) in-lab (*n* = 21) and (f) Exp. (2) online experiments (*n* = 90). Mean latencies of rod fixing in both environments are shown as grey dots. Line of no effect is shown as a dashed line. We predicted that most dots would lie above this line indicating slower action initiation for non-rewarding actions in the low value environment due to the lower opportunity cost. *t* statistic shows difference between mean log latencies, ** *p* < 0.01 *** *p* < 0.001.

The effect of OCT on mean latencies and free-operant action initiation is shown in Fig. 2 for both in-lab (Fig. 2a) and online samples (Fig. 2b). Mean latencies decreased as price of fish increased both experiments. Further, mean latencies were lower in the high OCT environment, in which ¥3000 was worth £4.00 as compared to £0.50 in the low OCT environment. Using mixed linear models to summarise the group level effect of both OCT manipulations on action latency, we found that participants in both Exp. (1) and Exp. (2) adapted their action latencies with respect to OCT for both price and environment. For both price [Exp. (1): *β* = −0.056, confidence interval (CI) −0.07 to −0.03, *p* < 0.001, Exp. (2): *β* = −0.039, CI −0.05 to −0.03, *p* < 0.001] and environment (Exp. (1): *β* = −0.049, CI −0.08 to −0.01 *p* = 0.001, Exp. (2): *β* = −0.041, CI −0.06 to −0.02, *p* < 0.001), as OCT increased, action initiation latency decreased. There was also a gradual drift towards slower latencies over the course of the experiment (Exp. (1): *β* = 6.9 × 10^−5^, CI 4.8 × 10^−5^ to 8.8 × 10^−5^, *p* < 0.001, Exp. (2): *β* = 4.0 × 10^−5^, CI 3.1 × 10^−5^ to 4.9 × 10^−5^, *p* < 0.001) in both experiments. Differences in total actions initiated between two environments is shown in online Supplementary Fig. S2.

In keeping with our predictions, participants in both studies also took longer to fix the broken fishing rod in the low OCT environment as compared to the high OCT environment (Exp. (1): *t*(20) = −3.0, *p* = 0.0076, Exp. (2): *t*(89) = −4.0, *p* < 0.001, Fig. 2c, d). Fixing the fishing rod was associated with no reward itself, other than a faster return to fishing.

### Individual sensitivity to opportunity cost predicted behavioural apathy scores in young adults

**Fig. 3.**
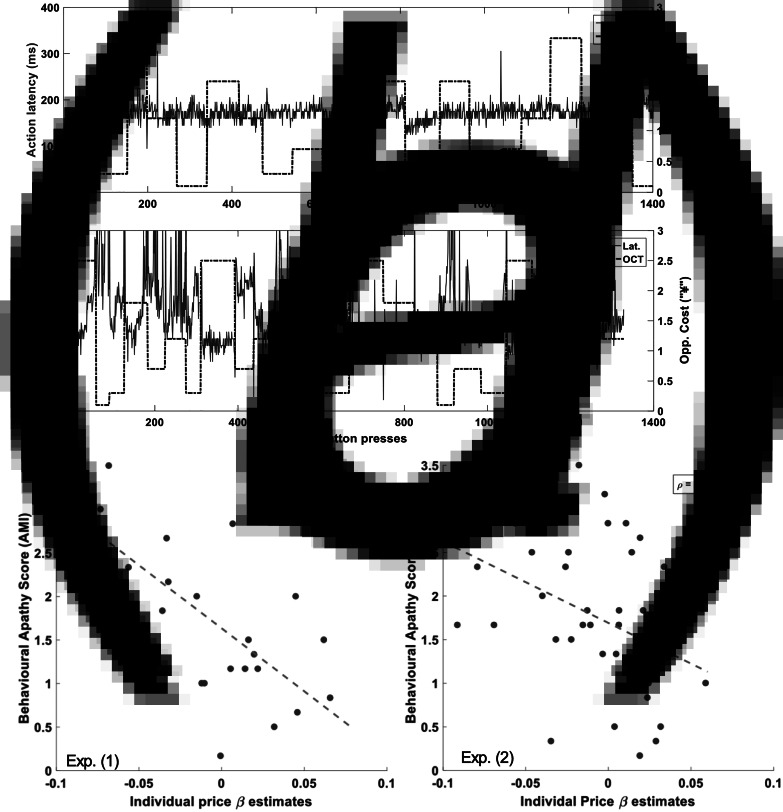
Sensitivity to opportunity cost depends on apathy – Example timeseries from the task as performed by a participant with low behavioural apathy (a – bAMI: 0.83) and a participant with high behavioural apathy (b – bAMI: 3.5) in Exp. (1). Grey unbroken timeseries shows chosen action latencies and broken lines indicate the current fish price. Changes in fish price signal change in OCT, here in the low OCT environment in both examples. Highly motivated individuals like the participant in (a) showed little sensitivity to change in opportunity cost. By comparison, the example apathetic individual in (b) showed a negative relationship between action latency and OCT. (c, d) Relationship between behavioural apathy scores measured by bAMI and OCT sensitivity (subject-level price *beta* from linear mixed model) in C. Exp. (1), in-lab (*n* = 21) and D. Exp. (2), online young adults (18–35 years, *n* = 45). Behavioural apathy scores were significantly associated with OCT sensitivity in both lab (*ρ* = −0.60, *p* = 0.004) and online samples (*ρ* = −0.50, *p* = 0.0005) in young adults. ** *p* < 0.01 *** *p* < 0.001.

As this task was designed to assess the effect of OCT on free operant action initiation, we hypothesised that sensitivity to OCT would predict behavioural apathy scores (bAMI). Figure 3a, b show example timeseries from two participants from Exp. (1) with low and high behavioural apathy scores, respectively. The high apathy individual showed a strong inverse relationship between latency and price. This effect was also seen at a group level. A linear regression model was used to assess whether participants' sensitivity to opportunity cost: either price or environment, could predict behavioural apathy scores after controlling for age, gender, and anxiety and depression scores. In Exp. (1), both price and environment sensitivity predicted behavioural apathy (Price GLM: *β* = −11.5, *t* = −3.1, *p* = 0.002, Fig. 3c, Environment GLM: *β* = −5.2, *t* = −2.2, *p* = 0.04).

We next tested whether apathy was correlated with both price and environment sensitivity in Exp. (2). Including the entire cohort (*n* = 90), we did not initially find the same relationship between behavioural apathy scores and price or environment sensitivity scores (Price GLM: *β* = −3.4, *t* = −1.7, *p* = 0.08, Environment GLM: *β* = 1.2 *t* = −1.6, *p* = 0.1). As compared to Exp. (2), all participants in the first in-lab study fell within the young adult age bracket, namely between 18 and 35 years. We therefore completed a *post-hoc* analysis dividing the cohort into young (age: 18–35, *n* = 45) and older adult cohorts (age: 36–65, *n* = 45) predicting that we would replicate the relationship between bAMI and price sensitivity in the young cohort. Replicating the results from Exp. (1), we found that price sensitivity predicted bAMI scores in young adults after correcting for age, gender, anxiety and depression scores scores (Young Adult Price GLM: *β* = −9.9, *t* = −3.2, *p* = 0.003, Fig. 3d). This result was not seen in the older adults (Older Adult Price GLM: *β* = 3.0, *t* = −1.2, *p* = 0.25). This result was not explained by a lack of effect of a price manipulation in older adults (Older adults: *β*_price_ = −0.041, *t* = −3.4, *p* < 0.001; *β*_env_ = −0.029, *t* = −1.5, *p* = 0.13; Young adults: *β*_price_ = −0.036, *t* = −7.4, *p* < 0.001, *β*_env_ = −0.053, *t* = −2.3, *p* = 0.018). Environment sensitivity was not predictive of behavioural apathy scores in either the young or old adults (Young Adults: *β* = 0.69 *t* = −1.4, *p* = 0.62, Older Adults: *β* = 1.6 *t* = 1.9, *p* = 0.06). In keeping with this, difference in rod fixing latencies and apathy did not correlate either (Exp 1: rho = −0.24, *p* = 0.3, Exp. 2: rho = −0.2, *p* = 0.18). In Exp. (1) price sensitivity predicted neither social apathy (*β* = −2.8, *t* = −0.7, *p* = 0.49) nor emotional apathy (*β* = −5.7, *t* = 1.3, *p* = 0.21) as measured by the sAMI and eAMI respectively. Replicating results from Exp (1), price sensitivity in Exp. (2) did not predict sAMI (*β* = −6.2, *t* = −1.8, *p* = 0.074) or eAMI (*β* = −1.2, *t* = −0.48, *p* = 0.64) scores in the younger adult cohort online. In summary, the sensitivity of young adults, tested either in the lab or online, to adapt their action latencies to changes in OCT (using the price manipulation in this task) predicts behavioural apathy.

### Reward sensitivity and OCT correlate with apathy scores in young adults

**Fig. 4.**
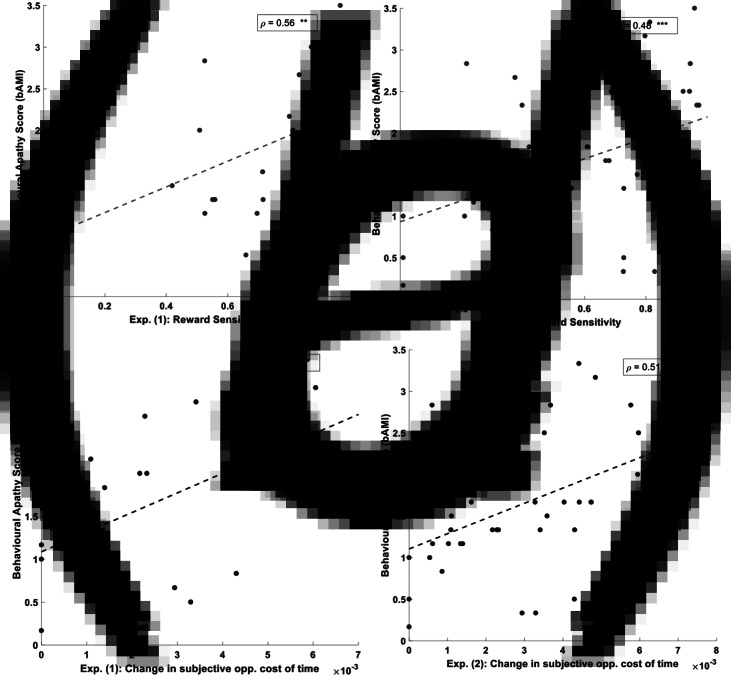
Apathy modulates reward rate: (a, b) We found a strong positive relationship between apathy and the reward sensitivity parameter in our average reward RL model. (c, d) As a result of this variation in reward sensitivity, apathetic individuals showed larger changes in subjective OCT derived from the model between different environments (plot shows the difference in modelled opportunity cost between states with the highest and lowest opportunity cost) *p < 0.05 ** *p* < 0.01 *** *p* < 0.001.

Using our average reward RL model, we found that reward sensitivity in young adults was correlated with independently assessed apathy scores in both Exp. (1) (*ρ* = 0.62, *p* = 0.008) and Exp. (2) (*ρ* = 0.52, *p* = 0.0009), Fig. 4a, b. In our model, a subject with high reward sensitivity would perceive large differences in subjective reward between prices and environments. By comparison, with low reward sensitivity, as found in motivated individuals the subjective reward remains high even when the price or environment reward value is low. As a result, our model also suggests that apathetic individuals in our studies showed the largest change in the subjective OCT throughout our task. This is demonstrated in Fig. 4c, d which show the correlations, in both Exp. (1) and Exp. (2), between individual behavioural apathy scores and the change in model-derived subjective OCT between states with the highest and lowest opportunity cost in our task.

Further, our modelling revealed that more apathetic individuals experienced higher OCT in the highest environment (online Supplementary Fig. S3a, b). This modelling result makes an intriguing prediction: more apathetic individuals will respond faster in the highest reward state (highest price and environment). We found that, in keeping with the modelling, higher apathy was surprisingly associated with a *lower* median action initiation latency in the highest reward state, with a negative correlation in Exp. (2) (*ρ* = −0.48, *p* = 0.0008) and a similar trend in Exp. (1) (*ρ* = −0.35, *p* = 0.1). These data are shown in online Supplementary Fig. S3a–d.

## Discussion

One of the hallmarks of apathy is reduced self-initiated goal-directed behaviour (Levy & Dubois, 2006; Marin, 1991). In this study, we show that healthy participants adapt the timing of self-initiation according to the OCT, the amount of reward lost per unit time by not acting. By manipulating the OCT within our novel behavioural task, we show in two independent studies that healthy participants rapidly adapt action initiation latencies for rewarding actions to changes in OCT. We also show that high OCT invigorates non-rewarding actions. Furthermore, we find that individual sensitivity to OCT predicted behavioural apathy scores in young adults in both studies. Building on these results we fit a novel computational model to behaviour in our task. Using our average-reward RL model we find that differences in reward sensitivity correlated with motivational status as assessed by a standard apathy questionnaire. This meant that apathetic young adults in our studies showed the largest change in the subjective OCT.

We believe that our study has several strengths. Firstly, the task we developed encourages free operant action initiation without adopting a discrete trial-by-trial design, making it highly ecological. Secondly, by making explicit the current reward rate, we minimised behavioural differences between participants driven by differences in learning. Furthermore, changes in OCT were signalled with salient visual and auditory stimuli to minimise the impact of inattention. As a result of these design elements, we contend that variance in the behaviour we observed is driven primarily by variance in the sensitivity of individuals to opportunity cost. Through these design elements, we also hope our task will be of value in a range of clinical populations. Following on from the first experiment, we also sought to independently replicate our results by running our second experiment online. By adopting this approach, and replicating our main results, we sought to avoid effects driven by any recruitment bias associated with laboratory cognitive testing or any demand effect due to the presence of the experimenter.

We would also like to highlight a few potential limitations of this study. Although we predicted a relationship between opportunity cost sensitivity and apathy, we did not *a priori* predict that this relationship would be influenced by aging. In our online study, older apathetic adults were not more sensitive to opportunity cost and this finding requires further investigation. This may reflect that fact that in the older adult cohort, the effects of the opportunity cost manipulations were weaker than in the young cohort. It is also known that aging influences a range of factors related to reward-based decision making and these changes may have contributed to our results (Chowdhury et al., 2013; Green, Myerson, & Ostaszewski, 1999; Rutledge et al., 2016). We also note that in this task, only price sensitivity predicted apathy and not environmental sensitivity (or rod fixing). We would not have predicted this dissociation as both price and environment represent manipulations of opportunity cost.

It may be argued that in our experiment reward cannot be dissociated from opportunity cost. As such, our experiment resembles previous work demonstrating harder work for more rewards – a well-known and well-characterised phenomena (Croxson, Walton, Reilly, Behrens, & Rushworth, 2009; Hamid et al., 2015; Manohar et al., 2015; Walton, Bannerman, & Rushworth, 2002). In response to this we draw the reader' attention to the inclusion of the rod fixing manipulation which dissociates immediate reward from opportunity cost. Participants fix the rod faster, not for immediate reward but to minimise the time spent away from accumulating reward, the opportunity cost. We should however point out that in the computational formulation opportunity cost does not represent a distinct psychological variable (such as ‘effort’) but rather is a *projection of subjective net reward in the time domain*. In this sense, the opportunity cost theory argues that we work faster for higher rewards because subjective reward translates directly into the time domain in the form of opportunity cost. We believe the opportunity cost drives both the fishing and rod fixing effects however, rod fixing most clearly dissociates immediate reward from opportunity cost in this experiment. It should also be noted that although participants showed an invigoration for rod fixing, this effect is not the same as the ‘general invigoration’ described by Niv et al. (2007). Firstly, the rod fixing is not truly goal-independent and secondly, participants did not choose when to fix the rod. As such rod-fixing differs from, for example, grooming more quickly when hungry. An alternative design would have been to introduce an optional additional action into the task for which participants were rewarded with some non-monetary reward however, standardising the choice of this option across participants would be challenging to achieve. We would also argue, that despite the difference between rod-fixing and invigoration as described by Niv et al. (2007), participants in this study lose time to collect rewards much like hungry animals who choose to drink as opposed to look for food. As such, rod-fixing in this study is invigorated for the same reasons, namely due to the increased opportunity cost between the two environments.

To our knowledge, our study represents the first demonstration that opportunity cost drives free-operant action initiation. In two important earlier studies it has been shown that in a trial-by-trial cognitive paradigm participants modulate reaction times based on experimentally controlled average reward rates (Beierholm et al., 2013; Guitart-Masip et al., 2011). However, those studies used cognitive paradigms and were unable to test whether opportunity cost drives free-operant action initiation because participants in both studies were prompted to act and additionally had to account for a speed-accuracy trade-off in their decisions. Similarly, Constantino (2015) and Le Heron et al. (2020) found that OCT drove the timing of foraging decisions however these were also not free-operant tasks (Constantino & Daw, 2015; Le Heron et al., 2020). Perhaps most relevant to our findings of a link to apathy was a null result recently reported by Kos et al. (2017), who identified in a sample of 39 young adults aged 18–40, a lack of relationship between self-initiation latencies and apathy (Kos et al., 2017). Participants initially were cued to respond, then asked to choose between two actions and were free to decide on the timing of their chosen action. Three key differences between our studies may explain the lack of association reported by Kos et al. (2017): responses were cued, the decision-making component also may affect latencies, and finally the OCT was not easy for participants to compute. Our task is similar to many problems faced in the natural world, and perhaps the key to our identification of a novel link to behavioural apathy.

We also present an average reward RL model for human free-operant behaviour. Using this computational approach, we find that low apathy young adults act as though they were experiencing a similar OCT across all conditions in our task. In comparison, high apathy young adults experienced the greatest change in subjective OCT. In both experiments, our computational model showed that reward sensitivity can explain the relationship between apathy and task performance. The reward sensitivity parameter governs the change in subjective reward as participants move between states with different levels of reward. As predicted, highly motivated (low apathy) individuals acted as if all rewards were subjectively highly rewarding and consequently, they were invigorated in all states. By comparison, apathetic individuals acted as if they found small rewards subjectively less rewarding, choosing only to act rapidly for larger rewards. Our computational modelling also revealed that participants with greater apathy had higher OCT in the highest reward state. This result led us to uncover an unexpected and intriguing aspect of our data: in the highest reward state, apathetic individuals on the whole acted more quickly than non-apathetic individuals. These findings suggest that in both laboratory and online young adult cohorts, the effects of apathy on attaining rewards may be overcome by reserving effort for high value environments, and this surprising result is worthy of further investigation. Finally, although average-reward RL models are not common in computational modelling, they make the argument that in large, ergodic, environments the long-run average of rewards can be used to optimise behaviour (Mahadevan, 1996). Although cognitive tasks are often short lived, psychological phenomena, such as motivational status and mood, are often conceptualised as extending over longer time periods (days or weeks). It may be that average reward signals, computed over various timescales, may be a useful framework for assessing and modelling these longer lasting phenomena.

Finally, although we did not test the biological basis of opportunity cost coding, as predicted by Niv et al. (2007), empirical work supports the idea that tonic mesolimbic dopamine signalling covaries with reward rate and motivational vigour (Hamid et al., 2015; Mohebi et al., 2019). Given the consistent links we find between behavioural apathy and sensitivity to the OCT in young adults, we would predict that young apathetic participants will show the greatest change in behaviour with the pharmacological manipulation of dopamine. On this basis we would also predict that this task would be sensitive to dopaminergic depletion seen in PD – with patients off dopaminergic medications showing a similar pattern of response to the younger apathetic participants in this study. Beyond dopaminergic changes, we also believe that these results have implications for patients with depression. Depression is associated with aberrant reward processing and global changes in psychomotor speed – typically resulting in slowing. Based on our results, we predict that, secondary to aberrant reward processing in depression, impaired or reduced opportunity cost may drive psychomotor changes seen in depression. It should be noted that although we describe some of our cohort as being apathetic – the apathy measured in our samples represents a normal degree of variation in motivation seen in the wider population. Although we would expect these relationships to be borne out in clinical samples, our participants did not have clinical apathy. There are also other variables which may have contributed, such as trait impulsivity. Overall, we believe that our task would be highly translatable to clinical populations with high levels of apathy

## Conclusion

Using a novel task and computational model, we find that OCT is an important determinant in the choice of free-operant action initiation latencies in healthy participants. We also establish, for the first time, a link between sensitivity to OCT and severity of behavioural apathy in two independent studies. Apathy is poorly understood and disabling, and clinical apathy is difficult to treat. Our results suggest that better understanding how the OCT is represented in the brain and how it influences action initiation may allow us to better understand apathy.

## Data Availability

Data and code will be made available on reasonable request.

## Supplementary material

For supplementary material accompanying this paper visit https://doi.org/10.1017/S0033291721003469. click here to view supplementary material
