# Supplementary material for: Opportunity cost determines free-operant action initiation latency and predicts apathy
Source: Psychol Med. 2021 Oct 12;53(5):1850–9. doi: 10.1017/S0033291721003469 (PMC10106307; doi:10.1017/S0033291721003469)
Supplement: Supplementary file 1 [file S0033291721003469sup001.docx]

**Supplementary Methods:**

**Changes for online version:**

The task online was almost identical to the one described in the Methods however a few differences were implemented online. The task was coded in JavaScript and hosted on Gorilla (<https://gorilla.sc/>). The only differences in the design matrix from the in-lab version were that the prices changed every 13 seconds with no jitter, and the number of taps required to fix the rod was increased to 8. At the beginning of the experiment, online participants read through the same detailed instructions as the in-lab version. They completed a brief training in which they were instructed to tap for 26 seconds during which the price changed after 13 seconds. All participants were then asked binary (True/False) questions about the task to test understanding (for example, “When the water is blue, ¥3000 is worth £4 – True or False). Finally, all participants underwent a sound check to ensure that they could hear the bell when the price changed. The sound check involved correctly identifying three animal sounds. Participants who failed the sounds check (n = 4) were immediately excluded. Aside from the 4 participants who were excluded for failing the sound check, 4 participants were excluded due to errors with data at rod breaking (missing data or more than 40 taps recorded rather than the 8 specified suggesting a data storage error for those participants). A further two participants tapped very slowly (less than 1 tap per second on average) and were excluded. In total, 90 participants were included in the online sample.

**Computational modelling details:**

Our goal is to provide a normative account of why certain participants modulate their latencies according to both the price and the environment, whilst others respond quickly in all situations. We formulate this as a cost-benefit decision-making problem in which participants must maximize their reward rates by responding quickly, but trade this off against the cost of responding quickly. We use this formulation to specify that the differences in response latencies between individuals is explained by different sensitivities to rewards. We can then relate these differences in reward sensitivity to measures of apathy.

Following Niv et al. (2007) we used an average reward reinforcement learning modelling framework to link vigour and motivation. The modelling is described in detail here are in Fig. S1. We consider the task to be a real-time cost-benefit decision-making problem, which repeats over and over again. A participant must choose how fast to initiate actions, in order to maximize her rate of rewards:

$\bar{R}=\frac{total reward}{time}$

For simplicity, we assume that each [*price of fish, environment*] condition is a separate state. A participant starts off in a state, chooses a latency and returns to the same state, and the process repeats. The problem is thus recurrent, with 12 distinct states in our task (6 prices and 2 environments). A subject cannot respond faster than their fastest motor latencies $\left( \tau_{min} \right)$ taken to be the fastest latencies above 100ms. This excludes spurious responses like slips of the finger. We assume that per response, the participant gains a reward of subjective value (r_subj_) but incurs two costs (Fig. S1A, B) (i) a calorific/energetic vigour cost (−C_v_/($\tau-\tau_{\min}$)) of responding quickly and (ii) an automatic OCT (−$\bar{R}\left( \tau\right)$). Following Niv *et al.* (2007), we assume the vigour cost is inversely proportional to the latency of responses, with its slope determined by the parameter C_v._ The vigour cost for a participant is defined relative to their motor latency, becoming much steeper when the participant responds near their motor latency. The OCT denotes the average reward foregone by responding at a particular latency: slower responses in a high reward environment are costlier as several highly rewarding actions could have been taken in that time. Finally, we assume that for each participant, objective reward (r_obj_: reward per action converted to £) is transformed to the subjective reward (r_subj_) via a concave utility function (Fig. S1C):

$$r_{\mathrm{subj}}=Rnorm [1-\frac{1}{f\left( r_{objective} \right)+\frac{1}{S_{R}}} ]$$

where S_R_ denotes how sensitive a subject is to changes in objective reward. Low S_R_ implies low sensitivity to objective reward and vice versa (Fig. S1C). Graphically, the parameter S_R_ shifts the utility curve laterally, so that the curve saturates more or less quickly. R_norm_ is a normalizing constant calculated as follows:

R_norm_ = 1 – 1/[*f( r_obj_max_) + 1/S_R_*]

This ensures that the subjective reward function saturates at 1 for all subjects, and thereby constrains the maximum subjective utility. We used the function ƒ(r_objective_) = 0.5*r_objective_ - 0.5, to obtain the best fits to the data across subjects, although our results are independent of the choice of this function.

Under these assumptions, we can compute the maximum of the net sum of rewards and costs for any given latency (τ). This maximal expected return Q*(s,τ) depends on three quantities: (i) the subjective value of the currently available reward (r_subj_), (ii) the cost of vigour (−C_v_/($\tau-\tau_{\min}$) ), and (iii) the OCT, in addition to the maximal value of the state to which the process returns. Further, we assume that latencies are generated (by policy $\pi\left( s,\tau\right)$) according to a softmax choice rule over the optimal Q-values:

$$Q^{*}\left( s,\tau\right)={max}_{\tau}\left[ r_{subj}-\frac{C_{v}}{\tau-\tau\min}+\overset{-}{R}\left( \tau\right)+{max}_{\tau^{'}}Q\left( s,\tau^{'} \right) \right]$$

$$\pi\left( s,\tau\right)\propto\exp(\frac{1}{Z}Q^{*}(s,\tau))$$

The optimal latency ($\tau^{*}={argmax}_{\tau}Q^{*}\left( s,\tau\right))$ is longer when the cost of vigour is higher, and shorter when the reward rate is high as given by:

$\tau^{*}=\sqrt{\frac{C_{v}}{\overset{¯}{R}*}}$ +$\tau\min$

Note that the optimal latency decreases hyperbolically with the reward rate. We fix Z = 1/100 to ensure identifiability of C_v_ and S_R_ parameters, and Gaussian-like distributions. All Q-values reported are thus, in effect, percentage Q-values.

We fit the latency distributions for each subject in two stages. We first fit the vigour cost parameter (C_v_) to the latency distribution for the highest price and environment condition. This comprises the condition when the objective reward and the subjective reward are both maximal. Since we assume that all participants have the same maximal subjective reward, fitting the model to this condition only allows C_v_ to explain variance in this condition across subjects. This ensures that the vigour cost was constrained per participant. We then assumed the reward sensitivity (S_R_) parameter to be free and fit the latency data across price and environment conditions using maximum likelihood, using the fmincon function in Matlab, initializing our optimization at different parameter values. We restricted reward sensitivity (S_R_) to lie between 0 and 1, and vigour cost (C_v_) between 0.01 and 100.

We also considered a model in which vigour cost was fixed to its median value across participants and only reward sensitivity was free. We compared models’ performance using Bayesian Information Criteria (BIC) scores between these two models. The model with parameters fit in 2 stages was preferred by BIC compared to the other model (Fig. S4). Parameters obtained from the model with the two-stage fit per subject were then correlated with apathy scores.

**Supplementary Figures:**

**
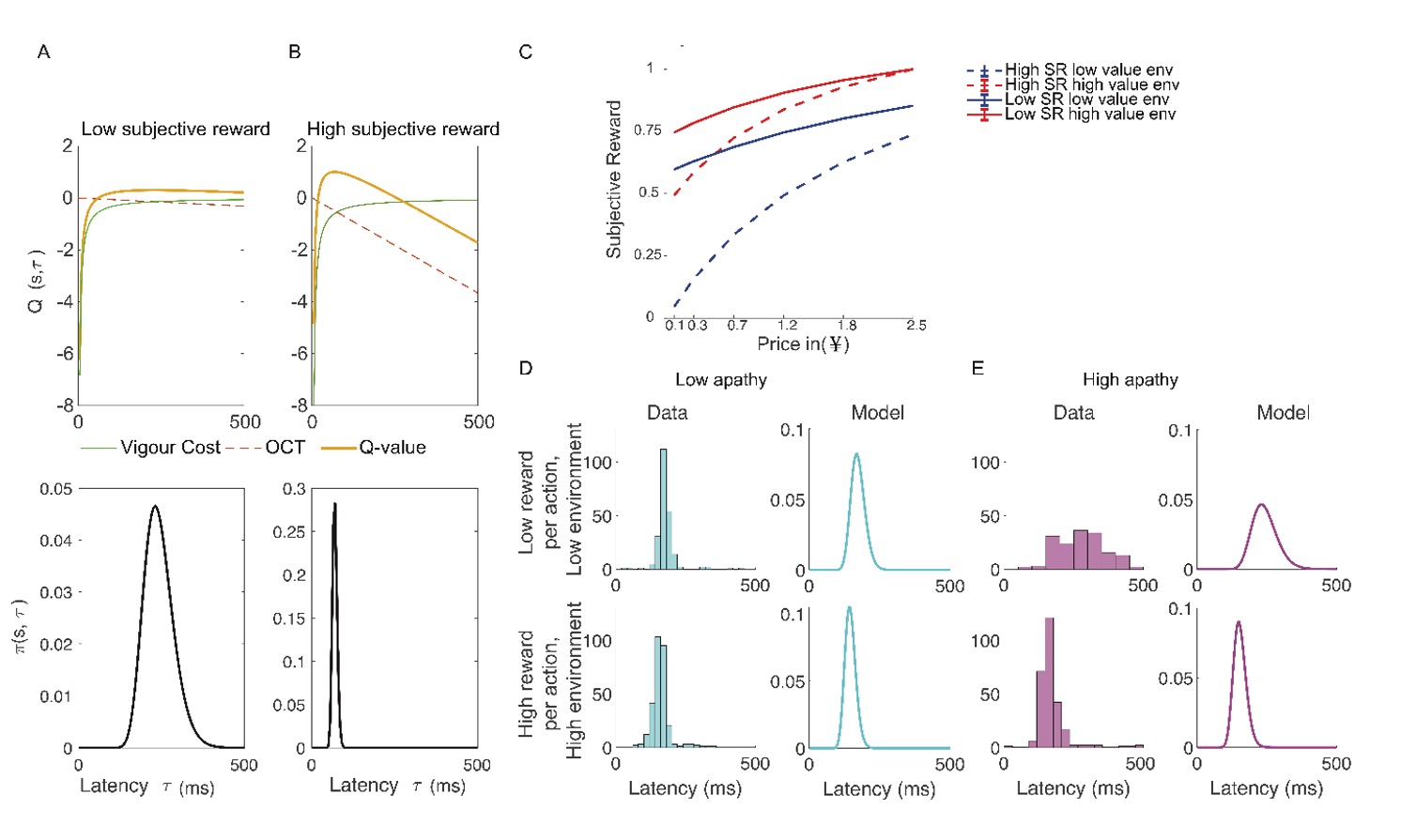
**

**Fig. S1: Average-reward reinforcement learning model captures action latencies** - (A,B) Model Mechanics. Each state *s* is defined as a [price of fish (in ¥), environment] condition. Upper panels: the net (*Q*) value of responding with latency (orange curve) involves trading off two quantities: the vigour cost (assumed to be inversely proportional to latency; green curve) and the automatic opportunity cost of time (OCT; linearly increasing with latency; red dashed line). Responding quickly reduces the OCT but incurs greater energetic/calorific vigour costs. The net (*Q*)-value curve, which is a summation of these two costs, is highest at the optimal latency. Lower panels: to generate a distribution of latencies, this *Q*-value is transformed with a softmax choice rule. (A) and (B) show the model’s mechanics for a low and high (subjective) reward, respectively. The OCT is greater when the subjective reward value per action for an individual is higher, shifting the *Q*-value curve leftwards and making the Q-value curve less shallow. Consequently, both the mean and variance of the latency distribution are smaller. (C) We transform each objective price of fish [(in ¥), for each environment] via a saturating concave subjective reward function. A subject with low sensitivity to reward (S_R_) should perceive little difference in subjective rewards between prices or environments. For such a subject, the subjective reward remains high even when the price or environment is low (solid lines). For a subject with high sensitivity to rewards, the subjective utility function will gradually increase with the price and environment. We assumed that the subjective rewards at the highest prices and environments would be the same for all subjects. (D-E) Latency distributions (left panels: data, right panels: model predictions) for the (D) lowest and (E) highest apathy in-lab participants, respectively. Top and bottom panels illustrate the response latencies for the lowest price of fish (in ¥) in the low-value environments and the highest price of fish in the high-value environments, respectively. For participants with low apathy scores, the latency distribution has a low mean and variance for both low and high rewards. For high apathy participants, the latency distribution shifts from having a large mean and variance to a small mean and variance. Our model predicts a similar relationship for subjects with low and high reward sensitivities respectively.


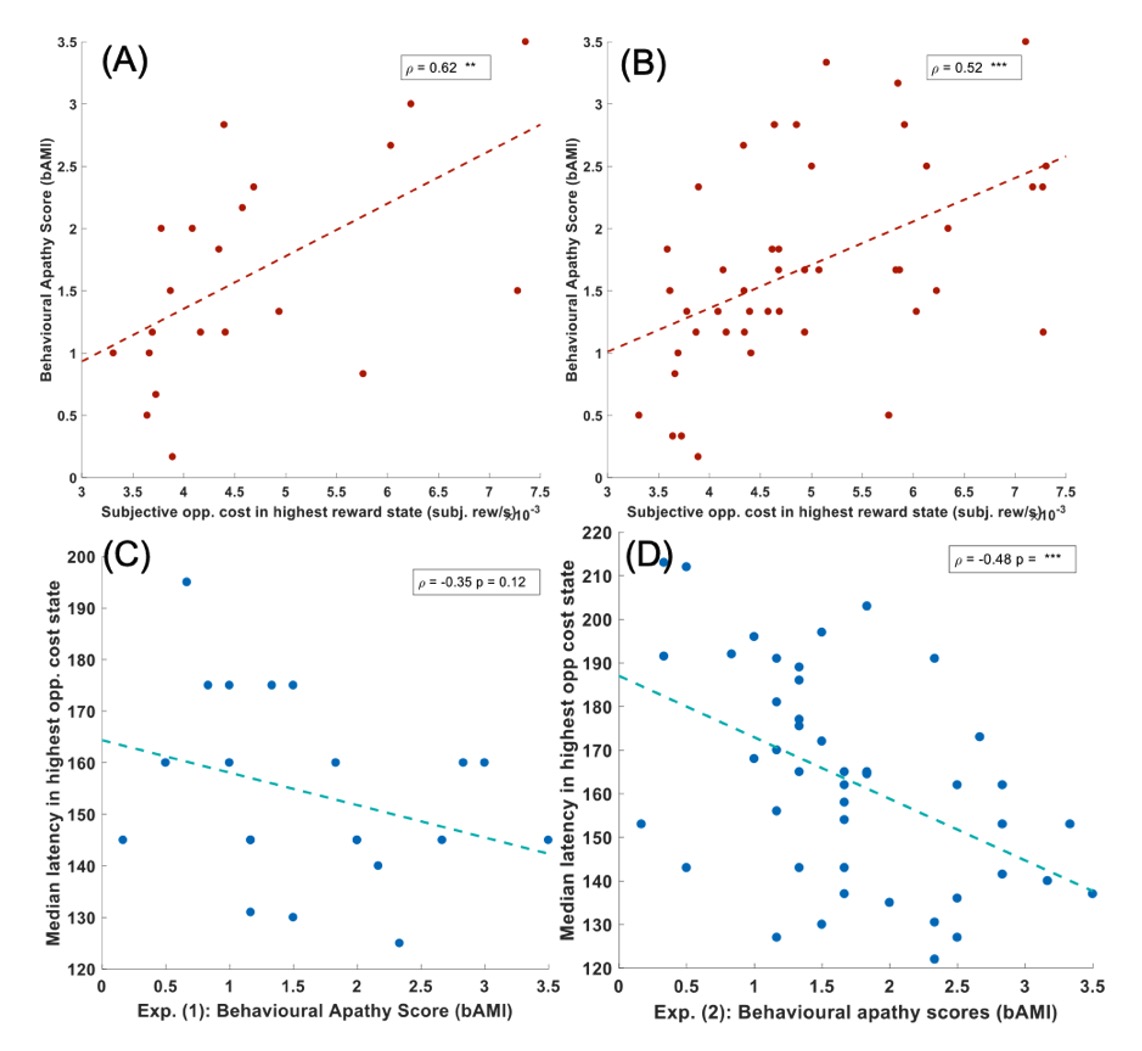


**Fig. S3:** A-B: association between the subjective opportunity cost in the highest reward state from our computational model and bAMI in Exp. (1) and Exp. (2) respectively. C-D: association between median action initiation latency and behavioural apathy scores in the highest rewarding state in Exp. (1) and Exp. (2) respectively. *p < 0.05 ** p < 0.01 *** p < 0.001


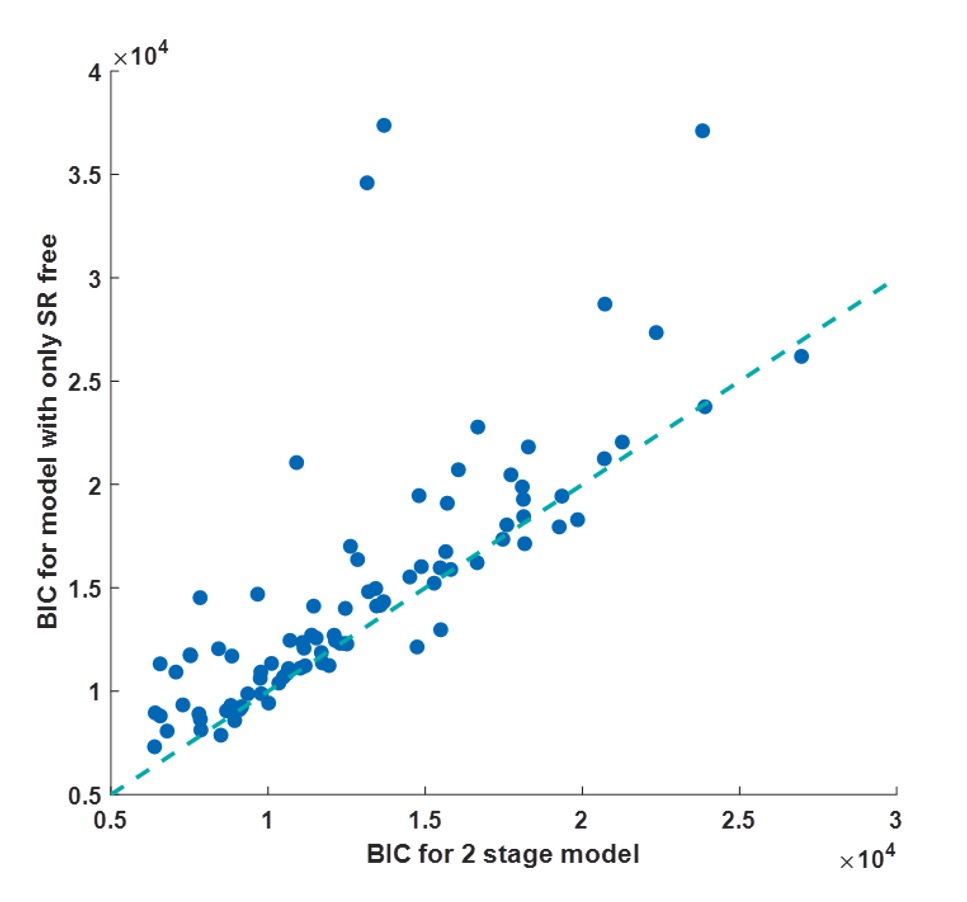


**Fig. S4:** model comparison showing the BIC for the 2-stage model (on the x-axis, in which Cv is fit in the highest reward state before S_R_ is then fit on the entire data) and a model in which Cv was constrained to the median value across all participants and then only S_R_ was fit (on the y-axis). Lower BICs indicate better model performance.
